# Supplementary material for: Developmental Unity and Cultural Variation in Forms of Metarepresentational False Belief Understanding
Source: Dev Sci. 2026 Jul 7;29(5):e70249. doi: 10.1111/desc.70249 (PMC13338850; doi:10.1111/desc.70249)
Supplement: Supplementary file 1 — Supplementary Table S1 Accuracy on control questions by task and cultural group. [file DESC-29-e70249-s002.pdf]

Supplementary Material S1
Control Questions: Structure and Performance

This document provides the exact wording of the control questions used in the tasks and summarizes participants' performance on these questions across communities.

Control Questions in False Belief Tasks

False belief tasks included two location/reality control questions (current location before and after the transfer).

- FB_CQ1: after first object placement: "Where is the [lemon]?"
- FB_CQ2: after object transfer, while protagonist is absent: "Where is the [lemon] now?"

Control Questions in Aspectuality Tasks

Aspectuality tasks included two parallel location/reality controls and an additional identity-ignorance control assessing whether the protagonist was unaware of the object's second aspect.

- Asp_CQ1: after first object placement: "Where is the [blue sock]?"
- Asp_CQ2: after revealing the dual aspect, while protagonist is absent: "Does [protagonist] know that the [blue sock] is also the [white sock]?"
- Asp_CQ3: after object transfer, while protagonist is absent: "Where is the [blue sock] now?"

Across all communities, children showed high accuracy on the control questions (see Table S1).

Table S1

Accuracy on control questions by task and cultural group.

Task	Culture	Correct_CQs	n	%
Aspectuality (pretense)	Germany	3/3	46	69.7
Aspectuality (pretense)	Germany	2/3	20	30.3
Aspectuality (pretense)	Hai‖om	3/3	58	92.1
Aspectuality (pretense)	Hai‖om	2/3	5	7.9

Task	Culture	Correct_CQs	n	%
Aspectuality (pretense)	Khwe	3/3	34	100.0
Aspectuality (realistic)	Germany	3/3	48	72.7
Aspectuality (realistic)	Germany	2/3	14	21.2
Aspectuality (realistic)	Germany	1/3	4	6.1
Aspectuality (realistic)	Hai||om	3/3	61	93.8
Aspectuality (realistic)	Hai||om	2/3	4	6.2
Aspectuality (realistic)	Khwe	3/3	35	100.0
False belief (pretense)	Germany	2/2	65	98.5
False belief (pretense)	Germany	1/2	1	1.5
False belief (pretense)	Hai||om	2/2	61	95.3
False belief (pretense)	Hai||om	1/2	3	4.7
False belief (pretense)	Khwe	2/2	35	100.0
False belief (realistic)	Germany	2/2	62	93.9
False belief (realistic)	Germany	1/2	4	6.1
False belief (realistic)	Hai||om	2/2	63	98.4
False belief (realistic)	Hai||om	1/2	1	1.6
False belief (realistic)	Khwe	2/2	36	100.0

Note. Correct_CQs indicates the number of control questions answered correctly.
